# Schizophrenia Induces Oxidative Stress and Cytochrome C Release in Isolated Rat Brain Mitochondria: a Possible Pathway for Induction of Apoptosis and Neurodegeneration 

**Published:** 2014

**Authors:** Mehrdad Faizi, Ahmad Salimi, Motahareh Rasoulzadeh, Parvaneh Naserzadeh, Jalal Pourahmad

**Affiliations:** School of Pharmacy, Shahid Beheshti University of Medical Sciences, Tehran, Iran.

**Keywords:** Apoptosis, Ketamine, Mitochondria, ROS formation, Schizophrenia

## Abstract

Schizophrenia is a chronic and often debilitating illness which affects about 1% of the world population. Some reagents have been used to simulate schizophrenic disorders in laboratory animals, such as amphetamine and ketamine. Previous studies have suggested that reactive oxygen species (ROS) production, reduced levels of ATP, mitochondrial dysfunction and apoptosis are involved in the pathophysiology and etiology of schizophrenia. In this study we divided Wistar rats in to 2 groups; control group received normal saline and test group received ketamine 30 mg/Kg daily for five consecutive days. Then, locomotor activity including side to side head rocking and arcing of neck, proved schizophrenia in the test group rats. Rats in both control and test groups were then decapitated and brain mitochondria were isolated. Our results showed increased ROS formation, mitochondrial membrane potential collapse, mitochondrial swelling and cytochrome c release in mitochondria of schizophrenic test group. Our findings suggested that mitochondrial ROS formation and apoptosis signaling are likely involved in cellular pathology of Schizophrenia. To our knowledge this is the first report that provides a mechanistic justification between mitochondrial events and neuodegeneration in the Schizophrenia.

## Introduction

Schizophrenia is a mental disorder characterized by a breakdown of thought processes and by a deficit of typical emotional responses (1). Prevalent symptoms include delusions and disordered thinking as auditory hallucinations, paranoia, bizarre delusions, disordered speech, and it is accompanied by significant social or occupational imbalances. The beginning of symptoms usually happens in young adulthood, with a prevalence of about 0.3–0.7% (2). Diagnosis is based on observed behavior and the patient’s reported experiences. There are many factors that can help to this mental disorder. These factors are genetics, neurobiology, environmental, psychological and social factors (3). Some recreational and nonprescription drugs for example amphetamine, cocaine, and alcohol, can cause or worsen schizophrenia symptom (4). The main treatment for schizophrenia is antipsychotic medications, which primarily decreases dopamine receptor activity. In many cases serotonin receptor activity also suppressed. Psychotherapy and vocational and social rehabilitation also must used in treatment of schizophrenia, because these factors contribute to faster treatment. In more serious cases involuntary hospitalization may be necessary (5).

Although cellular and molecular pathology and etiology of schizophrenia have not yet been elucidated, but devastated mitochondrial function and as a consequence impaired cellular energy state, is an attractive hypothesis for describing the pathophysiology of schizophrenia(6). Mitochondrion is a sub-cellular structure in the cell that has respiratory chain, that help to product energy. In some investigations, reduced levels of ATP were found in the frontal lobe, temporal lobe and basal ganglia of schizophrenic patients (7). Several genetic, neuron-imaging and clinical studies predicate mitochondrial dysfunction in schizophrenia. These studies shown that ROS formation at mitochondrial complex (I) is significantly increased in schizophrenic patients (6). Proteomics approach has been on human brain tissue to explore the molecular disease signatures. Many proteins identified by proteomics were associated with oxidative stress responses and mitochondrial dysfunction (8). Many studies have suggested that reactive oxygen species (ROS) production may play a role in the pathophysiology of many neuropsychiatric disorders, such as schizophrenia (9). 

There are a number of scrambles which want to explain the link between altered brain function and schizophrenia (2). One of these scrambles is the mitochondrial dysfunction (10). Some models have been used to simulate schizophrenic disorder, such as amphetamine and ketamine models (11). 

In this study we used ketamine for simulation of schizophrenic disorder in Wistar rat, and then we isolated mitochondria from brain of schizophrenic rat to figure out probable mitochondrial toxic mechanisms such as collapse of mitochondrial membrane potential, mitochondrial swelling, the raise of active oxygen radicals and finally release of cytochrome c which is the start point of cell death signaling.

## Experimental

*Animals*

Male Wistar rats (250-300 g) were housed in an air-conditioned room with controlled temperature of 25 ± 2 °C and maintained on a 12:12 h light cycle with free access to food and water. All experimental procedures were conducted according to the ethical standards and protocols approved by the Animal Experimentation Committee of Shahid Beheshti University of Medical Sciences, Tehran, Iran. All efforts were made to minimize the number of animals and their suffering.

*Drugs*

Ketamine was purchased from Sigma-Aldrich Co (Taufkirchen, Germany) and was dissolved in physiological saline. The drugs were intraperitoneally (*i.p*) injected at a volume of 1 mL/Kg body weight. All solutions were freshly prepared before injection. 

*Ketamine treatment*

Sub-anesthetic doses of ketamine induce psychotomimetic effects including a characteristic behavioral response consisting of staggered locomotion (12). Male Wistar rats were divided into two groups, each contains 7 animals. Rats were injected 30 mg/Kg *i.p. *ketamine for 5 consecutive days and rats in control group received normal saline at corresponding times.

*Locomotor activity test*

After five days of injection, locomotor activities including side-to-side rocking and arcing of neck were assessed according to previously described method in both control and ketamine treated test groups (13).

*Mitochondrial preparation*

After significant appearance of locomotor activities including side-to-side rocking and arcing of neck in ketamine treated test group rats, all animals in both test and control groups were sacrificed by cervical decapitation. Mitochondria were isolated from Wistar rat brain using differential centrifugation (14). Brains were minced and homogenized with glass hand-held homogenizer. The nuclei and broken cell debris were sedimented through centrifugation 1500×g for 10 min at 4 ºC and the pellet was discarded. The supernatant was subjected to a further centrifugation at 12,000×g for 10 min and the superior layer was carefully discarded. The mitochondrial pellet was washed by gently suspending in the isolation medium and centrifuged again at 12,000×g for 10 min. 

Final mitochondrial pellets were suspended in Tris buffer containing (0.05 M Tris-HCl, 0.25 M sucrose, 20 Mm KCl, 2.0 mM MgCl2, and 1.0 mM Na2HPO4, pH of 7.4) at 4 °C, except for the mitochondria used to assess ROS production, MMP and swelling, which were suspended in respiration buffer (0.32 mM sucrose,10 mM Tris, 20 mM Mops, 50 μM EGTA, 0.5 mM MgCl2, 0.1 mM KH2PO4 and 5 mM sodium succinate), MMP assay buffer (220 mM sucrose, 68 mM D-mannitol, 10 mMKCl,5 mM KH2PO4, 2 mM MgCl2, 50 μM EGTA, 5 mM sodium succinate, 10 mM HEPES, 2 μM Rotenone) and swelling buffer (70 mM sucrose, 230 mM mannitol, 3 mM HEPES, 2 mM tris-phosphate, 5 mM succinate and 1 μM of rotenone). Protein concentrations were determined through the Coomassie blue protein-binding method as explained by Bradford, 1976 (26). The isolation of mitochondria was confirmed by the measurement of succinate dehydrogenase (27) .Mitochondria were prepared freshly for each experiment and used within 4 h of isolation and all steps were strictly operated on ice to guarantee the isolation of high-quality mitochondrial preparation.

*Determination of mitochondrial ROS level*

The mitochondrial ROS measurement was performed using the fluorescent probe DCFH-DA. Briefly, isolated brain mitochondria were placed in respiration buffer containing 0.32 mM sucrose, 10 mM Tris, 20 mM Mops, 50 μM EGTA, 0.5 mM MgCl2, 0.1 mM KH2PO4 and 5 mM sodium succinate (15). Following this step, DCFH-DA was added (final concentration, 10 μM) to mitochondria and then incubated for 10 min. Then, the fluorescence intensity of DCF was measured using Shimadzu RF-5000U fluorescence spectrophotometer at an excitation wavelength of 488 nm and emission wavelength of 527 nm.

*Determination of the MMP*

Mitochondrial uptake of the cationic fluorescent dye, rhodamine 123, has been used for the estimation of mitochondrial membrane potential. The mitochondrial fractions (0.5 mg protein/mL) were incubated with 10 μM of rhodamine 123 in MMP assay buffer (220 mM sucrose, 68 mM D-mannitol, 10 mM KCl, 5 mM KH2PO4, 2 mM MgCl2, 50 μM EGTA, 5 mM sodium succinate, 10 mM HEPES, 2 μM Rotenone). The fluorescence was monitored using Shimadzu RF-5000U fluorescence spectrophotometer at the excitation and emission wavelength of 490 nm and 535 nm, respectively (16).

*Determination of mitochondrial swelling*

Analysis of mitochondrial swelling after the isolated mitochondria (0.5 mg protein/mL) was estimated through changes in light scattering as monitored spectrophotometrically at 540 nm (30 °C) as described (17). Briefly, isolated mitochondria were suspended in swelling buffer (70 mM sucrose, 230 mM mannitol, 3 mM HEPES, 2 mM tris-phosphate, 5 mM succinate and 1 μM of rotenone). The absorbance was measured at 549 nm at 10 min time intervals with an ELISA reader (Tecan, Rainbow Thermo and Austria). A decrease in absorbance indicates an increase in mitochondrial swelling.

*Cytochrome c release assay*

The concentration of cytochrome c was determined through using the Quantikine Rat/Mouse Cytochrome c Immunoassay kit provided by R and D Systems, Inc. (Minneapolis, Minn). Briefly, a monoclonal antibody specific for rat/mouse cytochrome c was pre-coated onto the microplate. Seventy-five μL of conjugate (containing monoclonal antibody specific for cytochrome c conjugated to horseradish peroxidase) and 50 μL of control and test group were added to each well of the microplate. One microgram of protein from each supernatant fraction was added to the sample wells. All of the standards, controls and test were added to two wells of the microplate. After 2 h of incubation, the substrate solution (100 μL) was added to each well and incubated for 30 min. After 100 μL of the stop solution was added to each well; the optical density of each well was determined through the aforementioned microplate spectrophotometer set to 450 nm.

*Statistical analysis*

Results are presented as mean ± SD. Assays were performed in triplicate and the mean was used for statistical analysis. Statistical significance was determined using the student t-test or one-way ANOVA test, followed by the post-hoc Tukey test when appropriate. Statistical significance was set at P < 0.05.

## Results

**Figure 1:**
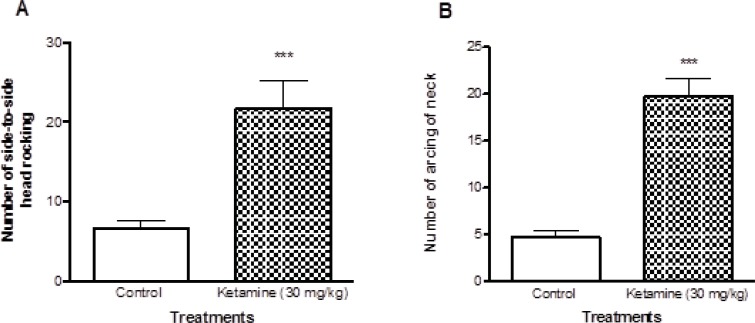
Comparison of locomotor activities between ketamine (30 mg/Kg *ip *× 5 days) treated and control rats. There were 7 rats in each group. A shows the number of Side to side head rocking behaviors and the B shows the number of arcing behaviors of neck in both ketamine (30 mg/Kg *ip *× 5 days) and control rats. Values are presented as mean ± SD (n = 7). *** represents *P *< 0.001 significant difference in comparison to control

The characteristic behavioral responses including side-to-side head rocking and arching of the neck were evaluated. Ketamine treated rats showed significant increase (P < 0.001) in both side-to-side head rocking and arching of the neck compared to control rats (Figure 1).

**Figure 2:**
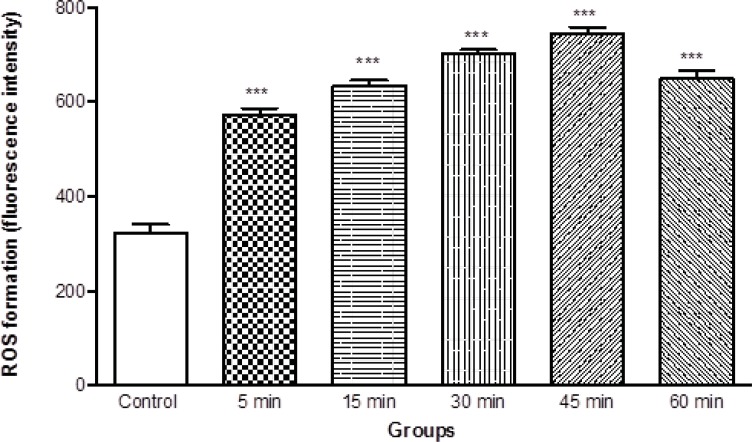
ROS formation in brain mitochondria isolated from ketamine (30 mg/Kg *ip *× 5 days) treated and control rats. ROS formation was measured fluorometrically using DCF-DA as described in materials and methods. Values are presented as mean ± SD (n = 3). *** represents *P *< 0.001 significant difference compared to control

As shown in Figure 2, ROS generation was significantly (*P *< 0.05) increased in isolated brain mitochondria obtained from ketamine (30 mg/Kg *i.p*. × 5 days) treated rats compared to control rats. The ROS formation in two groups was measured in the time intervals (15, 30, 45 and 60 min) following isolation of rat brain mitochondria in both groups.

**Figure 3:**
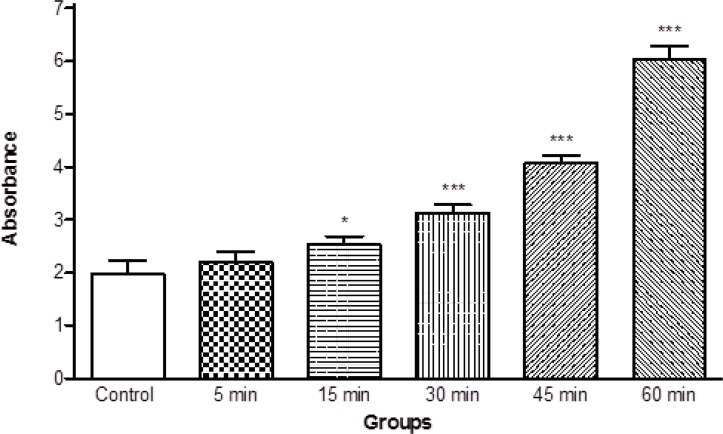
Progressive mitochondrial swelling in the brain mitochondria isolated from ketamine (30 mg/Kg *ip *× 5 days) treated rats compared with those of control rats. Mitochondrial swelling was measured through the determination of absorbance at 530 nm as described in Materials and methods. Values represented as mean ± SD (n = 3). * represents *P *< 0.05 and *** represents *P *< 0.001 significant difference compared to control mitochondria

Moreover, we determined the mitochondrial swelling, an indicator of mitochondrial membrane permeability (MPT), by recording the decrease of absorbance of isolated mitochondria obtained from ketamine treated rats at 530 nm (A540). Our results showed significant mitochondrial swelling (p < 0.05) in the mitochondria isolated from brain of ketamine treated test group compared with control group (Figure 3). The mitochondrial swelling in two groups was measured in the time intervals (5, 15, 30, 45 and 60 min) following suspension of isolated rat brain mitochondria in the swelling buffer.

**Figure 4:**
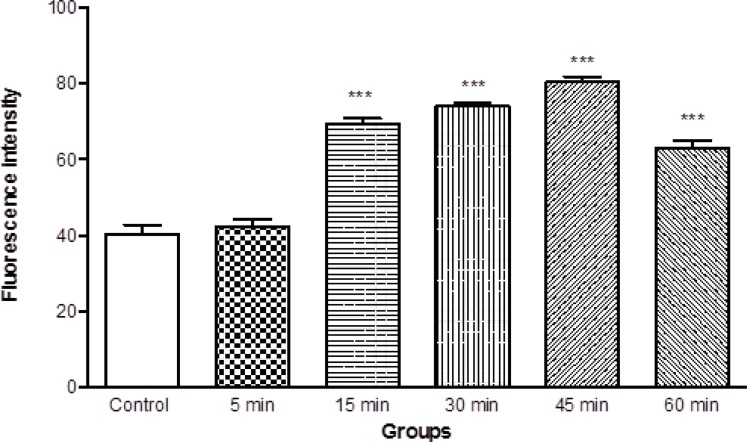
Mitochondrial membrane potential decrease in the brain mitochondria isolated from ketamine (30 mg/Kg *ip *× 5 days) treated rats compared with those of control rats. MMP was measured by rhodamin 123 as described in Materials and methods. Values represented as mean ± SD (n = 3). *** represents *P *< 0.001 significant difference compared to control mitochondria

Mitochondrial membrane potential is a highly sensitive indicator of the mitochondrial inner membrane condition was measured by Rhodamine 123 redistribution. As shown in Figure 4, MMP significantly (p < 0.05) decreased in the mitochondria isolated from brain of ketamine treated test group compared with control group (Figure 4). The mitochondrial membrane potential in two groups was measured in the time intervals (5, 15, 30, 45 and 60 min) following suspension of isolated rat brain mitochondria in the incubation buffer.

**Figure 5:**
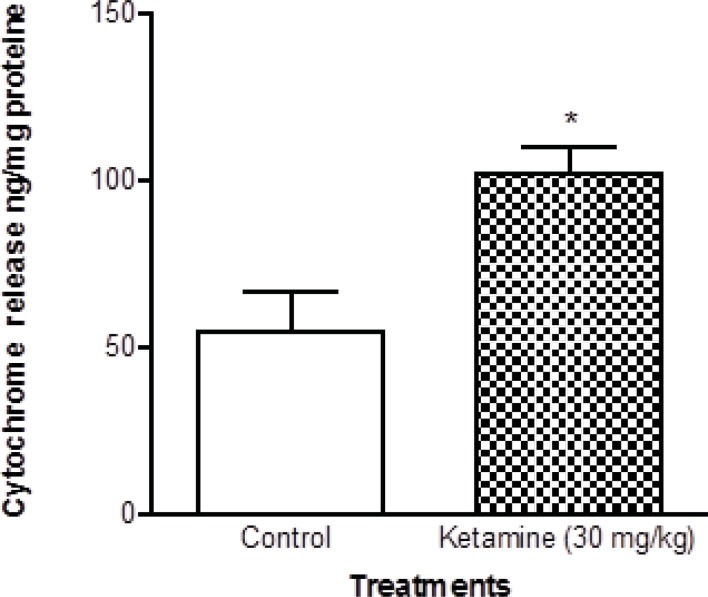
Cytochrome c in the brain mitochondria isolated from ketamine (30 mg/Kg *ip *× 5 days) treated rats compared with those of control rats. Cytochrome c release was measured by ELISA kit as described in Experimental. Values are presented as mean ± SD (n = 3). *:Significant difference in comparison with control mitochondria (p < 0.05).

Cytochrome c release, the endpoint of mitochondrial toxicity, was significantly (p < 0.05) increased in the mitochondria isolated from brain of ketamine treated test group compared with control group (Figure 5).

## Discussion

Schizophrenia is a chronic and often debilitating illness which affects about 1% of the world population. In addition to severely disrupting the life of the patient and their family, schizophrenia incurs a great cost to society in terms of lost productivity and treatment-related expenses. Although the etiology remains unknown, converging lines of evidence, including reduced gray matter volume, reduced synaptic markers and reduced neuropil, suggest that disrupted cortical synaptic circuitry is a central deficit in schizophrenia (18). The underlying mechanisms that lead to synaptic dysfunction are also uncertain; however, evidence suggests that a dysregulation of neuronal apoptosis, a form of programmed cell death, may contribute to the pathophysiology of schizophrenia (19, 20).

Some models have been used to simulate schizophrenia, such as amphetamine and ketamine. In this study we induced schizophrenia by ketamine and used (30 mg/Kg × 5 consecutive days) ketamine for stimulation of schizophernia in the rats. Then we assessed the locomotor activities including side to side head rocking and arcing of neck in two groups of ketamine treated and unexposed control rats. Our results showed that the locomotor activities in the ketamine treated group were significantly higher than control group (Figure1 and Table 1).

Apoptotic cell death can be mediated via several pathways. One pathway involves the mitochondria. Mitochondria are semi-autonomous organelles that play essential roles in cellular metabolism and programmed cell death pathways. Many diseases induce cellular stress, which may also lead to mitochondrial perturbation and, finally, cell death. Mitochondrial dysfunction is usually resulted from mitochondrial membrane permeability transition, dissipation of the inner membrane potential, osmotic swelling of the matrix, rupture of the outer mitochondrial membrane, release of cytochrome c and other apoptogenic proteins from the mitochondria, and formation of the caspase-3 activation complex, the apoptosome (21). Permeability transition involves the opening of a channel named permeability transition pore complex (MPT pore). The main components of this pore are adenine nucleotide translocator and cyclophilin D in the inner membrane of the mitochondria, and voltage-dependent anion channel and peripheral benzodiazepine receptor in the outer mitochondrial membrane.

There are a number of scrambles which want to explain the link between altered brain function and schizophrenia (2). One of them is the mitochondrial dysfunction (10). Our data suggested that in schizophrenia, mitochondrial ROS formation in CNS neurons causes MPT, leading to the collapse of mitochondrial membrane potential, mitochondrial swelling and cytochrome c release. The latter is the starter of apoptosis signaling. The release of cytochrome c from isolated mitochondria would slow down the electron transfer from complex III to complex IV and therefore enhance the ROS generation at the Q-cycle (22). In other words, ROS generation can occur even earlier than cytochrome c release from mitochondria. This may explain the puzzle between the ROS generation and cytochrome c release, which is usually considered a delayed event (23). 

The increased mitochondrial ROS formation can cause oxidation of a lipid membrane results in disruption of the mitochondrial membrane and consequently the collapse of mitochondrial membrane potential (MMP) and cytochrome c release. Previous studies showed that MMP represents the integrity of the mitochondrial membrane and its metabolic activity as a key indicator of mitochondrial functionality (24). Furthermore, MMP is a crucial factor for the regulation of mitochondrial activity and the MMP collapse is the important stimuli for apoptosis (24). 

Altered regulation of the apoptotic cascade can potentially reduce neuronal and glial viability at various stages of neural development and contribute to the volumetric and functional brain deficits observed in schizophrenia (20).In particular, apoptosis has been identified as a potential underlying mechanism for evidence of progressive gray matter volume loss seen around the first onset of psychosis (21). Our results also provided evidence that mitochondrial ROS formation and apoptosis signaling are involved in cellular pathology of Schizophrenia. To our knowledge this the first report that provides mechanistic reasons between mitochondrial events and progressive brain neuronal loss through apoptosis in the Schizophrenia.
